# The Hospital. Nursing Section

**Published:** 1903-09-26

**Authors:** 


### The Hospital.

### Nursing Section.

Contributions for this Section of "The Hospital" should be addressed to the Editor, "The Hospital" Nursing Section, 28 & 29 Southampton Street, Strand, London, W.C.

No. 887.—Vol. XXXIV. *SATURDAY, SEPTEMBER 26, 1903.*

### Notes on News from the Nursing World.

### MR. CHAMBERLAIN AND COLONIAL NURSING.

The members of the Colonial Nursing Association will deplore the resignation, by Mr. Chamberlain, of the office of Colonial Secretary. He was in office when the Association was formed, and, in fact, it was largely due to the sympathy and assistance which he readily afforded to Mrs. Piggott, that she was able to carry into execution her carefully thought-out scheme. Of course the special feature of the Colonial Nursing Association is that it is the accredited source whence the Colonial Office draws its supply of matrons and nurses for Government hospital appointments in the Crown Colonies; and Mr. Chamberlain has always been most appreciative of the work done under its auspices, recognising in the most cordial manner its practical importance and value. It has been a source of strength to the managers to know that they could at all times feel that he regarded the development of the Association with lively interest and satisfaction.

### THE FICTION THAT NURSES ARE SHORT-LIVED.

We give the exceedingly interesting and important circular issued to the policy-holders of the Royal National Pension Fund for Nurses, *in extenso*, in another page, because we believe that a great many of our readers who, not being members, will not receive a copy, will be impressed by the points which have been summarised from the supplementary report furnished by the consulting actuary in connection with the quinquennial valuation. The report should have the effect of largely dispelling a fiction which is still widely prevalent. It would hardly be an exaggeration to say that the majority of members of the nursing profession are possessed by the idea that nurses are short-lived persons, although if they were asked their reasons, they would find it difficult, if not impossible, to give them. The recent valuation of the Pension Fund proves once more that the reverse is actually the case, and that, instead of nurses "dying young," they are, as a body, longer-lived than women in other walks of life. Thus, Mr. King, in his report, states that if the annuitants had been women in other walks of life, the anticipated number of deaths during the last five years would have been 32, while, as a matter of fact, there were only 23. Again, the actual number of deaths amongst the holders of sickness assurance policies was 21, while according to the Government annuity tables, the number expected was 70. The moral of such figures as these is obvious. If they conclusively dispose of the notion that those who pursue the vocation of nursing die early, they emphasise, in a striking manner, the need for nurses to make provision for old age. It must be remembered that in addition to their chances of living longer lives than the average woman, the years during which they are able to obtain remuneration for their work are fewer. This should be an incentive to all who have not joined the Pension Fund to seriously consider their prospects. We can quite understand the disinclination of young nurses to begin saving. But a nurse who from the outset makes it a rule to set aside a certain portion of her income for the purpose of insuring herself against poverty or misfortune, cannot fail to find it much easier to pay the premium of the Pension Fund than the nurse who postpones taking out a policy until she has got into the habit of spending all her earnings as soon as she receives them.

### SHIPPING COMPANIES AND THE NURSING QUESTION.

We understand that so far only two of the shipping companies have returned a definite answer to the proposal that they should employ nurses on board their ocean-going steamers. These, we are sorry to learn, were refusals; but it is to be hoped that the silence of the other companies implies that the proposal is being seriously considered. The addition of a trained nurse to the staff on a liner would involve a trifling increase in expenditure, but the owners, we believe, would find compensation in the augmented confidence of the public. That there would be no lack of eligible candidates for such posts is shown by the fact that since the name and address of Miss Penn, who has taken up the matter, appeared in our columns, she has received nearly 30 applications, including two from matrons, who are anxious to know the outcome of the movement.

### THE RECENT LIBEL ACTION BY A HOSPITAL NURSE.

Referring to the recent action for libel against Miss Loveys, the lady superintendent of the Queen Victoria Nursing Institution, Wolverhampton, we understand that, at the monthly meeting of the committee of the institution, held last Friday, Miss Loveys was the recipient of a cheque for £355 and an album. The inscription in the album is as follows:—"Presented to Miss Loveys, matron of the Queen Victoria Nursing Institution, together with a purse of £355, as a mark of the respect and esteem in which her personal character and work are held by the friends whose names are inscribed in this album." The Chairman, Mr. G. N. Adams, J.P., in presenting the testimonial, spoke of the energy and ability displayed by Miss Lowe, the honorary secretary, in soliciting subscriptions towards this laudable object; although Miss Lowe had reached more years than the allotted span, she achieved her object within a fortnight. The testimonial was a token of sympathy with Miss Loveys in the loss she sustained in a recent action, and it was felt that the opportunity should be given to her many friends to participate in it. The chairman and committee subscribed most generously, and the appeal on all sides met with a most hearty response. There are 80 names in the album in addition to those of the nursing staff of the Queen Victoria Institution and the matron and nursing staff of the District Nurses' Home; and they embrace all the representative people of Wolverhampton and the neighbourhood.

### AN INCIDENT AT AN ISOLATION HOSPITAL.

The nurses at the Billericay Isolation Hospital have been called over the coals because they refused to receive a patient suffering from enteric fever, and sent him to the Union Infirmary. Dr. F. Carter, the medical officer, explained that only one of the two women at the isolation hospital was on duty at a time, that there was a scarlet fever case, and that it would not be right for a nurse to be left alone with a man who might become delirious. Moreover, he added, Dr. Hilliard had sanctioned the reception of the patient in the Union Infirmary. This being so, the subsequent remark of the chairman of the Billericay Board of Guardians that he "did not approve of nurses being the masters of the guardians," was as uncalled for as his contention that "a good many persons in the workhouse were endangered by the reception of a patient suffering from enteric fever in the infirmary" was absurd. It is a matter for satisfaction that the nurses knew what was the proper course to pursue in the circumstances.

### VISITING NURSES IN MANCHESTER.

The managers of the Manchester and Salford Nursing Institution have taken an important step. They have determined to try to meet the wants of a large section of the community in Manchester requiring the services of a trained nurse by a system of daily visits at greatly reduced rates. For a single visit by a nurse, who in all cases will be fully qualified, the charge is half-a-crown, and chronic cases needing two or three visits a week will be attended for half a guinea a month. For those who may desire a nurse to attend for some time, a daily visit a week is offered at a charge varying from 5s. 6d. to half a guinea a week according to the nature of the case, and two daily visits a week will be made for a fee varying from 10s. to £1 a week. In Manchester, as in London, there are many cases, notably of aged patients, who, while not ill enough to demand the constant presence of a nurse, ought to have occasional attendance, but who go without it because shillings have necessarily to be considered. The result of this experiment will therefore be watched with interest.

### STARTING A HOME.

At the sixth annual convention of the Nurses' Associated Alumnæ of the United States, an address was delivered by Miss E. R. Scovil, of the Massachusetts General Hospital Alumnæ, upon "The Private Hospital as Owned and Managed by Nurses." She mentions as the first requisite a small capital of from one to two thousand dollars (£200 to £400 roughly), according to the size of the contemplated hospital, and, if possible, not borrowed capital. Having chosen a locality she advises the nurse to personally visit the doctors whom she wishes to interest in her project, to select a house with a number of smaller rooms rather than a few large ones, and to furnish them very simply but with fresh and dainty articles. She considers it wise for two or three nurses to club together and start a home, as it lightens work and expenses, and then to enlist the services of a mother or sister—not a trained nurse—to do the housekeeping and see that the food supplied to the patients is the best of its kind. All charges, Miss Scovil urges, should as far as possible be inclusive, unless a special nurse be required, and all patients should be asked to pay weekly in advance. She adds that a nurse who can give massage would find she had a special advantage in an institution of this sort, and that a maternity home is sometimes much wanted in certain localities. All this is interesting, but so far as starting a home in England is concerned, we warn nurses that the capital suggested by Miss Scovil is utterly inadequate. It must be assumed that at least three years' maintenance will have to be provided, and this means quite £1,500, to which should be added the cost of equipment, another £700 or £800. Even with ample capital, and with the recommendations of doctors assured, there is a serious risk of losing money in a nursing home, and the best advice we can give the average nurse is to leave it alone. Perhaps the value of our advice might be emphasised by the relation of the personal experience of some of our readers, and we therefore invite any who have started, or tried to start, a home, to send us a brief account of their success or failure.

### THE RELIGIOUS QUESTION AT TYRONE INFIRMARY.

An interesting discussion on the religion of the nursing staff at Tyrone County Infirmary is reported in the Irish press. It is a matter for regret that such discussion should be considered necessary, but the committee of management of the Tyrone Infirmary may be congratulated upon the temperate manner in which they treated a topic that so often unhappily excites acrimonious controversy. The point at issue was whether a statement made by Monsignor McNamee, that undue preference was given by the governors to Protestant nurses, was correct. Dr. Thompson dealt at length with this allegation, and while admitting that the number of Roman Catholic nurses in the hospital was not equal to that of Protestants, succeeded, we think, in proving that this was not due to any religious prejudice on the part of the governors, but simply to the fact that the proportion of suitable applicants for posts who were Protestants had been much larger. Dr. Thompson himself, Sir John Stewart, and other speakers, expressed a desire that in view of the Roman Catholic patients in the Infirmary being nearly two to one against Protestants there should be a much larger proportion of Roman Catholic nurses, and Father O'Kane said that he had never heard a word of complaint against the matron. But when applications are made the governors act rightly in refusing to ask a candidate her religion, and in voting for the one who has the best testimonials of capacity.

### THE HOSPITAL-TRAINED ASYLUM MATRON.

The appointment of matron of the male department of the Stirling District Asylum, Larbert, has been accepted by Miss Irvine, who is a fully-certificated hospital nurse. In addition to gaining her certificate for general training at the Western Infirmary, Glasgow, she was afterwards sister for two years and a half in Sir William T. Gairdner's wards. Her experience as a mental nurse has been gained at Perth District Asylum, and on the completion of her training there she became matron of the Royal Asylum, Dundee. She therefore, like her predecessor, Miss Wise, comes to her new post fully equipped. The multiplication of hospital-trained nurses as matrons, who have also received mental training, is one of the means to which we look for the elevation of the status of the mental nurse.

### STRANGE SITUATION AT LADYWELL WORKHOUSE.

It has been pointed out to us that the Ladywell workhouse, to which we alluded in our issue of the 12th, is not an infirmary, but "a workhouse for the aged." Strictly speaking this may be the case, but unless there are a number of sick persons in the institution, why do the authorities regularly employ a superintendent nurse, and a head night nurse, as well as an assistant matron, who is also a trained nurse? If there is plenty of work for these three officials, how can the institution be equally well worked when two of the three—in addition to the master and matron—are away at the same time? Moreover, if the committee believed that things could go on quite as satisfactorily with four officers absent simultaneously, why was an application made—although unsuccessful—for a nurse to Guy's Hospital Nursing Institution? It is not suggested that the doctor, the assistant matron, and the attendants did not do their best for the women who died, but it stands to reason that in an institution with 718 inmates, many of them patients, four important officers cannot all be away together without a serious danger of duties of some kind being neglected.

### THE GRANARD SCANDAL.

The Granard Board of Guardians have postponed consideration of the letter of the Irish Local Government Board in which they dismiss the master of the workhouse, condemn the employment of Dr. Maguire, and censure Dr. Dolan, making a record against him in the books of the department, until their next meeting in October. But it it does not auger well for the fulfilment of the hope expressed by the Local Government Board that, "the guardians will now endeavour to settle all outstanding questions with regard to the nursing in the workhouse," that "a unanimous vote of confidence" in the dismissed master has been passed by the guardians.

### DISTRICT NURSING IN SWANSEA.

Although the café chantant in the grounds of Llwynderw on behalf of the Swansea District Nursing Association was held under the most unfavourable auspices so far as weather was concerned, the whole of the entertainments having to be given in the home instead of outside, there was a goodly attendance and an enjoyable afternoon was spent. Lady Lyons, who opened the proceedings, made an excellent little speech in which she traced the history of the district nursing movement in the town. She strongly recommended that the working classes of Swansea should be canvassed with the view of inducing them to support the association by regular contributions, however small; and she emphasised the importance of this point by mentioning that she had been told that if every working man in the town gave a penny a week, the managers would have enough money to carry on the work satisfactorily. There are now three nurses, but a fourth is badly wanted. We trust that the receipts at the café chantant—an excellent means of raising money in summer—will materially benefit the organisation.

### PROGRESS AT FAVERSHAM.

The Faversham Board of Guardians, after a conference with the medical officer, have, we are informed, decided to have a female nurse and a female assistant on the male side of the infirmary, in place of the male nurse who recently resigned. They have also determined to appoint the matron of the workhouse, who is a trained and certificated nurse, to the post of superintendent nurse with an additional salary of £10 per year, and to allow the nurses the sum of £5 annually for uniform.

### SHIRLEY WARREN INFIRMARY.

The nurses at the Shirley Warren Infirmary appear to have misunderstood the decision of the Southampton guardians, for they have memorialised the Board to rescind the resolution, "forbidding the matron and medical superintendent to give testimonials." We should agree with the nurses if this determination had been arrived at, that they would have a difficulty in obtaining posts of responsibility; but as it was pointed out at a subsequent meeting of the guardians, the form of testimonial is to be filled up by the medical officer and matron, and signed as approved by the Board. The nurses, in these circumstances, do not seem to us to have any cause for protest, and in face of a recent incident, we should have concluded that they would hail the course determined upon by the guardians with satisfaction.

### A STORM IN A TEA-CUP.

The Bristol Board of Guardians, whose inability to decide whether their Hospital Committee should be allowed to possess the power of requiring a probationer to resign, was the subject of comment a fortnight ago, have at length arrived at a conclusion. At the last meeting of the Board there was another protracted discussion, but in the end an attempt to refer the question to the Finance and General Purposes Committee was defeated by a majority of 10 votes. The Hospital Committee are not therefore under an obligation to resign, and the Local Government Board will not be asked to intervene in a dispute in which too much time has already been spent.

### SHORT ITEMS.

Miss E. S. Mason, Miss M. M. Rees, and Miss M. Walker have been appointed staff nurses, provisionally, and Miss E. M. Monck-Mann sister, provisionally, of Queen Alexandra's Imperial Military Nursing Service.—At a Baby Show in South London last week the judges, three doctors, were assisted in the discharge of their duties by Nursing Sister Lemon and the Ladies' Committee.

### The Examination of the Urine.

By J. K. WATSON, M.D.

DESCRIPTION OF THE URINE (*continued from page* 301).

V. *Reaction.*—Healthy urine has usually an acid reaction, but after a meal it may be alkaline. After it is passed, urine generally undergoes a change in its reaction, at first becoming more acid, but finally becoming alkaline. These changes are due to fermentation set up by micro-organisms. But urine may be alkaline in its reaction constantly, and the most common cause of this is inflammation of the bladder (cystitis), and is very apt to arise in such cases from the use of a dirty catheter, as it also occurs in some forms of paralysis.

VI. *Odour.*—Healthy urine has a slight characteristic odour. Alkaline urine has very often a smell of ammonia, which is, as we have said, due to alkaline fermentation, whereby the urea is transformed into carbonate of ammonia. The urine under such conditions is termed ammoniacal, and it is usually very offensive. In diabetes mellitus the sugar gives to the urine a sweetish smell, said to resemble the odour of apples or of new-mown hay.

The presence of acetone in the urine also produces a fruity smell. Certain drugs, such as turpentine and cubebs, produce a characteristic odour, the former causing an odour of violets. Asparagus, too, produces a peculiar odour in the urine after it has been eaten.

VII. *Deposit.*—There is seldom any deposit in healthy urine, although a brick-red sediment, signifying the presence of urates, sometimes occurs in cold weather, especially after prolonged exercise, which cannot then be considered abnormal. The cloud of mucus which we have mentioned, may come to form a deposit. Phosphates are sometimes deposited in the urine. This usually signifies insufficient exercise with sedentary occupation, a febrile condition, nervous exhaustion or actual disease of the nervous system. Urates or, as they are sometimes termed, lithates,[1] are only deposited on cooling of the urine. When passed, the urine is clear. Gastric derangements, great exertion and fever are responsible for their deposit. They are of little consequence unless they persist. Uric acid may be deposited as a "cayenne-pepper" sediment. Pus may be deposited either as pure pus or mixed with mucus (muco-pus). It resembles phosphates at first sight, but on tilting the glass the phosphatic deposit is not so compact and, being more easily separated, tends to float up from the bottom of the glass.

VIII. *The Solid Constituents of the Urine.*—We now pass to consider the composition of the urine, first referring to the normal and afterwards to the abnormal constituents. The daily average amount of solids passed in the urine is usually stated to be 950 grains, or, if we use the metric system about 60 grammes (1 gramme = about $15\frac{1}{2}$ grains). A useful and fairly accurate way to determine the solids is to take the specific gravity and multiply the last two figures by the number of ounces passed in the 24 hours, and then to multiply the result by $1\frac{1}{10}$ or, using decimals, by 1·1. The following will serve as an example:—A man passes 46 ounces of urine in the 24 hours having a specific gravity of 1018. How much solids does he pass in the urine in 24 hours? Applying our formula we get $46 \times 18 \times 1{\cdot}1 = 910{\cdot}8$ grains, which amounts to just over two ounces. Such a method is not applicable to urine containing albumin or sugar or other abnormal ingredients. There are a considerable number of solids in the urine, which we may divide into organic and inorganic, the former being derived from the animal and vegetable worlds the latter from the mineral world. The most important constituent of all and that which represents the chief waste matter of the body is a substance called urea. It represents about one-half of the total solids of the urine and about 500 grains are excreted daily. Urea is very soluble. It is found in the blood and in nearly every tissue of the body, from which it is extracted by the kidneys. The more nitrogenous, that is to say animal, food we eat the more urea do the kidneys remove from the body up to a certain point. But even when no nitrogenous food is taken, some urea is excreted in the urine; this shows us that the urea is derived from the waste that goes on in the tissues as well as from the food. The quantity of urea is affected by disease; in fevers and in diabetes mellitus it is increased in amount, in the various forms of Bright's disease it is diminished. We have already mentioned the pigments of the urine which must be included in the organic constituents. The only other organic solid requiring mention is uric acid, which does not usually occur in normal urine as the free acid, but is combined with potash, soda and ammonia to form salts known as urates. About ten grains (or one-fiftieth of the amount of urea) leave the body daily on an average. The chief inorganic constituents of the urine, in addition, of course, to water, are chlorides, sulphates, phosphates (which, as we have seen, may form a deposit), and, to a slight extent, oxalates.

1. The first of the abnormal constituents is albumin. The term albuminuria is used to express the presence of albumin in the urine. Albumin may occasionally be present in health (functional albuminuria); but, in nearly every instance, at any rate where it persists, it indicates a diseased condition. It is not easy to explain why, under certain conditions, albumin passes into the urine, but, probably, in the vast number of cases it is due to the lining (epithelium) of the urinary tubules failing in their "selective" action, whereby water and various salts are allowed to pass the barrier, but other substances such as albumin are held back. Albumin most commonly occurs in the urine in acute and chronic kidney disease (Bright's disease), in certain forms of heart disease, especially when accompanied by dropsy, in certain fevers such as scarlet fever (in the later stages where the kidneys are apt to become affected), diphtheria, and in certain nervous and in some chronic general affections such as anæmia. It must be understood that albuminuria is not necessarily present in all these diseases; very often it is absent, sometimes throughout the course of the illness, if we except cases of Bright's disease, in which the urine always contains albumin at some period of the illness. It will be well here to refer to what are known as tube casts which are often found in kidney disease. These may be regarded as moulds, more or less complete, which have been washed away from the walls of the inflamed tubes by the urine. These tube-casts are sometimes the only positive evidence of kidney disease. They can only be detected microscopically unless very numerous, when they may form a sediment at the bottom of the urine glass, which can be seen by the naked eye. The more important kinds of casts are epithelial, granular, hyaline (transparent and colourless), and blood, the last-named being due to blood, which has been poured out into the tubules, becoming coagulated.

2. The next abnormal ingredient is blood, its presence in the urine being signified by the term hæmaturia when all its ingredients are passed; when the pigment without the corpuscles is passed the term hæmoglobinuria is used. The former is by far the commoner condition. When blood or pus are present in the urine albumin in greater or less amount is necessarily present as blood and pus both contain

[1] Greek lithos, a stone.

THE EXAMINATION OF THE URINE—*Continued.*

albumin. When blood is in small amount it gives to the urine a peculiar opaque appearance which is generally described as "smoky"; in larger amount it produces a red colour depending for its intensity on the amount of blood present. Blood may be present in the urine as a result of a variety of conditions, and it may be derived from the kidney, the ureter, the bladder, or the urethra. In the last-named situation the blood is nearly always passed with the first part of the urine, the latter portion of the urine being free from blood. Blood derived from the kidney is usually well mixed with the urine. If the blood is seen to be in long, thin cylindrical clots, it has probably come from the pelvis of the kidney, and has been moulded in its passage by the ureter.

3. *Sugar.*—We have already seen that sugar is present in the urine in cases of diabetes mellitus. The form of sugar which is passed is not, however, the cane sugar to which we are accustomed, but another kind of sugar named "glucose," "grape sugar," or "dextrose." The presence of sugar in the urine is generally referred to as glycosuria. It should be understood that although the presence of sugar in the urine in the large proportion of cases is due to diabetes mellitus, yet it is not always so due. In fact, very slight traces of glucose do occur in healthy urine, but so slight is the amount, that it cannot be detected by the tests which we shall describe (see Part III.). We must regard, therefore, the presence of sugar in the urine as due to a fault in the transformation of the food substances whereby sugar comes to circulate in the blood. The only part the kidneys play in this connection is that of removing this sugar from the blood through the medium of the urine.

4. Bile may be found in the urine. This substance is poured out by the liver and passes into the first part of the small intestine (duodenum). When, from any cause, bile circulates in the blood a condition known as jaundice arises, and bile is separated from the blood by the kidneys, and passes out in the urine. The colouring matter of the bile is known as bilirubin and it is this which gives to the urine in such cases its yellowish-brown colour. After standing, the bilirubin becomes changed to another substance called biliverdin, which produces a greenish tint. Bilious urine becomes frothy on shaking.

5. Pus in the urine has been mentioned when speaking of deposits. It may occur in many conditions. The commonest are abscess of kidney or an abscess bursting into the urinary tract, inflammation of the bladder (cystitis), where it is generally combined with mucus (muco-pus), gonorrhœa, and leucorrhœa (in women), commonly called "white discharge" or "the whites."

(*To be continued.*)

### The Royal National Pension Fund for Nurses.

THE QUINQUENNIAL VALUATION.

THE following circular has been sent to the policy-holders of the Royal National Pension Fund by the Secretary, Mr. Louis Dick, at the instance of the Council:—

"You will remember that in the last annual report a supplementary report was promised as soon as our consulting actuary, Mr. George King, F.I.A., F.F.A., had completed his investigations; the calculations have been even more laborious and complicated than on former occasions, so that this report has only recently reached the Council's hands.

"As the report contains a great deal which is of a technical nature, the Council have thought it advisable to condense it into the form of this circular.

"The following are the principal points:—

"POLICIES IN FORCE.—On December 31st, 1902, there were 7,161 contracts (policies) in existence, securing annuities to the extent of £115,705 19s. 1d. per annum.

"ANNUITIES.—Annuities were being paid under 610 policies.

"PROFIT BONUS.—The liabilities amounted to £601,459 8s., while the funds in hand were £629,404 0s. 1d., showing a surplus of £27,944 12s. 1d.; this means that a profit of that amount was made during the five years ended December, 1902, and that such profit was more than two and a half times greater than in 1897.

"DONATION BONUS.—The accumulation of interest available for distribution on the same basis as that adopted in former valuations was £8,621 7s.

"The Council have decided to distribute £26,000 of the profit (carrying forward the balance to the next valuation) and the whole of the amount available for donation bonus as on former occasions, making a total sum at their disposal of £34,621 7s.; in 1897 the amount so distributed was £16 952, consequently, a sum of over £17,600 in excess of 1897 is now being distributed.

"The following table will be of interest as showing the extraordinary growth of the Fund during the last fifteen years; the figures given relate to the five years ending December 31st of 1892, 1897, and 1902 respectively:—

| | 1892. | | | 1897. | | | 1902. | | |
|---|---|---|---|---|---|---|---|---|---|
| Single premiums received | £16,236 | | | £38,242 | | | £78,371 | | |
| Periodical premiums received .. .. .. | 68.793 | | | 181.886 | | | 299 500 | | |
| Interest and dividends .. | 7,418 | | | 35.861 | | | 89,763 | | |
| Annuities paid .. .. | 521 | | | 4.863 | | | 24,183 | | |
| Surrender values paid .. | 4.850 | | | 33.665 | | | 89.215 | | |
| Returns of premium .. | 376 | | | 1.884 | | | 9,248 | | |
| Funds in hand .. .. | 126.490 | | | 377 800 | | | 734,182 | | |
| Profit bonus .. .. | Nil. | | | £10.303 | 18 | 9 | £26 000 | 0 | 0 |
| Donation bonus .. .. | £3,227 | 10 | 5 | 6.648 | 5 | 3 | 8,621 | 7 | 0 |
| Total .. .. | £3,227 | 10 | 5 | £16,952 | 4 | 0 | £34,621 | 7 | 0 |
| Policies in force at end of quinquennium .. .. | 2,412 | | | 4,964 | | | 7,161 | | |

"The valuation establishes beyond question the fact that the premium which a nurse pays into the Fund produces a very much better annuity than that obtainable under any other circumstances.

"After 15 years' working the Fund has been able (exclusive of the special bonus to the 'first thousand' nurses) to distribute £54,801 1s. 5d. amongst its policy-holders with the object of increasing their annuities when they become due, a result which even the most sanguine of its friends could hardly have anticipated at its foundation.

"The Council heartily congratulate all policy-holders on the results disclosed by the valuation. The individual profit bonus will be higher than at the last valuation, and this, in spite of the fact that the holders of policies who joined prior to 1898, being five years older, a larger sum had to be put by to provide a bonus which should merely be equal to that of 1897.

"There is one feature in Mr. King's report to which the Council wish to direct the special attention of policy-holders and of nurses generally, namely, that the valuation of the Fund once more establishes the fact that nurses as a body are long-lived—longer lived, in fact, than women in other walks of life—and this is a point which nurses ought to have brought forcibly before them, the more so, because there is undoubtedly an idea prevalent that the reverse is the case, and that nurses die young. Mr. King states that if the annuitants had been average women, meaning women in ordinary walks of life, the number of deaths during the last five years would have been 32, while, as a matter of fact, there were only 23.

"Amongst the holders of sickness assurance policies, the number of deaths was 21, while, according to the Government annuity tables, the number of deaths expected was 70. Although these figures go to prove that nurses are long-lived, the fact must not be overlooked that their working years, or, more correctly, perhaps, the years during which they are able to obtain work, are in reality fewer than those of other women; consequently, they have a longer period to bridge over, during which they will only be supported by the provision which they have made for themselves, consequently it behoves them to begin early and to continue adding to such provision.

"It is as well to remind you that the object of the Fund being to give a nurse as large an annuity as possible, the principle of the distribution of bonus is that she shall benefit by the additions when she enters upon her pension. Bonuses, therefore, are not distributed in cash, or applied to the purpose of reducing the premiums.

"While every policy issued by the Fund entitles its holder to bonus additions, the individual bonus addition is only communicated to policy-holders after the valuation which follows the completion of five years from the issue of the policy; if, therefore, your policy was issued before January 1st, 1898, you will be advised as to the amount of the addition if you will apply in writing (a postcard will be sufficient), to the office, 28 Finsbury Pavement, E.C."

### How Shall I Become a Nurse?

BY AN EX-MATRON.

First of all ascertain your physical fitness for nursing work. Go to a medical man and ask him to examine you, and should he consider you strong enough to bear the strain of hospital life, to give you a certificate to that effect. Your family doctor, who has perhaps known you all your life, might be the best man to consult, but do not try to bias him by making the granting of a good health certificate a personal favour. Impress upon him that you wish for his candid opinion, and that, should it be unfavourable to your joining the nursing profession, you are prepared to give up the idea. If you go to a doctor who knows little or nothing about you, be sure to tell him all your weak points. For instance, if you have pains after food, are subject to headaches, find your eyesight or hearing defective, however slightly, tell him. Remember the certificate to be worth anything must guarantee not only that you are at present in good health, but that you are a healthy woman, free from organic disease or weakness, and in full possession of your five senses. The certificate obtained, make some half-dozen copies and put them into envelopes. The original put carefully away until it is asked for. Next have your photograph taken, carte-de-visite size, three-quarter length, write your name and the date on which it was taken on the back, and put one into each envelope with the certificate. Direct these envelopes to yourself at a permanent address, put on a penny stamp but do not close them. Do not dress up for the photograph. Be done in your ordinary dress with your hair as you usually wear it.

### The Next Step.

Then, having ascertained your exact weight and height (without shoes), take in The Hospital weekly and answer all the advertisements that you think would suit you; do not be too particular, more especially if, as is often the case, you cannot afford to pay a premium, or even to work for a year or two without remuneration. Remember that even in small provincial hospitals, where a salary is given, there are endless applicants for every vacancy. At the same time it is useless to apply for posts that you would not care to accept. It is difficult to give advice as to how your application should be worded, so much depends on what is required on both sides, but I think the following is a good sample of what is usually required, and could easily be altered to suit circumstances:—

Dear Madam,—Having seen your advertisement for a Probationer I beg to apply for the post. I am 26 years of age, height 5 ft. 4 in., weight 9 stone. My father is (or late father was) a (here mention occupation of parents) and I (here state your religion). I have lately been employed as a governess, and can give you a reference to a lady, with whom I have lived three years, and whom I am only leaving to be trained as a nurse. I enclose copy of health certificate and a recent photo, which I should be much obliged to have returned as soon as possible. I shall be at liberty in about a fortnight's time. Should you consider me a suitable applicant would you kindly forward me your rules and regulations for nurses.

I remain, yours faithfully,

### No Omissions.

If there is anything you think detrimental to your being accepted, such as being a little above or below the age specified, do not think that omitting to mention the fact will help you over the difficulty. It will in all probability only cause your application to be put aside in favour of some other that gives the necessary information. If you do not get a reply at once, do not be disheartened. There are many reasons, too lengthy to mention here, that may prevent applications from being answered for some days, or even weeks. In the meanwhile you need not waste your time. If you have not been successfully re-vaccinated within the last year or two, be done. Go to a dentist and have your teeth put in perfect order; don't say "I never have any trouble with my teeth." Make sure that you cannot have trouble with them. So important is this that many hospitals, before engaging probationers require a dentist's, as well as a doctor's, certificate.

### The Dress Question.

Then provide yourself with two quite plain print dresses (they need not be new), made just to touch the ground, and with the sleeves arranged so as to be easily turned up to the elbows. A few bib aprons (calico ones will do), a pair of ward shoes, with broad toes and flat heels, stocking suspenders instead of garters, scissors, a pocket pincushion filled with ordinary and safety pins, half a dozen sets of white collars and cuffs, and all the special outfit you will probably require to begin your new work is ready. One thing more you can do with much advantage. At the Nurses' Club, 12 Buckingham Street, Strand, are held classes especially intended for probationers about to enter hospital life. There, for the small fee of 10s. 6d., you can learn elementary anatomy, bandaging, and such nursing work as taking temperatures, keeping charts, measuring and administering medicines, reading prescriptions, medical signs, etc., and many other things a previous knowledge of which will be most helpful to the new probationer when the time comes to enter the hospital ward.

### Be Always Ready.

One more piece of advice. Once you are accepted on the staff of what you hope soon to speak of as "my hospital" remain at the address you have given the authorities. More especially in small hospitals, where the nursing staff does not allow of a margin for emergencies, vacancies are apt to occur unexpectedly, which must be filled at once. Many a probationer has been saved weeks of weary waiting because she was the only one on the matron's list who could come at a few hours' notice. Hold yourself in readiness to be that one, and you will have made a good start in the nursing world.

### Sketches of Our Hospital:—The Dining Room.

BY E. MARGARET FOX.

Sunlight everywhere. An irregular hexagon, the room is so built that three of its sides are nearly all lofty windows, facing the morning sun. The dew is yet on the broad green lawn outside, and a silvery web runs across one of the open windows. The hospital cat is perched on a sill, and eyes the sparrows burrowing in the flower-beds under the big "Monkey" tree, with covetous orbs, dilated with greed. Tall green palms are on the two long tables laid for breakfast. The varnished wall-paper and the paint of the woodwork are all toned in a restful green, and the [floor is stained a deep brown. The long window-curtains and blinds are of a green that harmonise well with the rest of the room. A clock, a notice-board, plain Windsor chairs, a "baby" harmonium, two bookshelves, a sideboard, and the tale of furniture is complete.

Seven o'clock strikes, and a gong sounds almost simultaneously. Presently, the nurses come into breakfast, in little groups of twos and threes, arm linked in arm, dropped decorously at the door, some bright as the morning itself, some with the night's sleep not yet well out of their eyes. Five minutes' grace is allowed: then the door is shut, and any who enter afterwards are counted "late." There is a great scramble during that last five minutes. Cuffs are fastened, and caps pinned, on their way downstairs from the distant dormitories, and aprons are being tied during their rapid flight through the corridors. These, of course, are the tardy ones, and not those who hold the envied reputation of never being late; but soon all are gathered in the spacious dining-room, and breakfast is well on its way. Their faces are an interesting study as they sit there. One of them —pale, thin, ascetic-looking—makes you instinctively feel how entirely in its right surrounding it would be framed by a broad white wimple, its owner wearing the black habit of a nun, instead of the lilac gown of a nurse.

The next is a great contrast. Brown, laughing eyes, straying chestnut hair, that from its disposition to wave and curl calls forth an occasional reproof from the authorities, a bright, sunshiny, cheery, lovable little face, that does the patients good by its very brightness. The round cheeks are still sun-kissed from a recent holiday, and the plump hands brown as berries. She chats away blithely to an older nurse at one end of the table, who is pouring out tea. This one has a rugged, sensible face, full of sound judgment and certain trustworthiness. There is much penetration in the keen brown eyes, and a latent gleam of humour round the mouth. She says little, and that little with great deliberation. The element of impulsiveness is altogether absent, and she can be most surely trusted to tread without a hair's breadth's deviation from the old tried paths she knows so well. She is a nurse to be valued in these go-ahead days of erratic impulses and ambitious tendencies, and her face has been a familiar one at our hospital for many a year. To the young probationers she is at once a curb and an encouragement. They have a great respect for her, and they know that it is better to have her opinion of them favourable rather than adverse.

A face of Madonna-like sweetness is the next, truth and sincerity gazing at you from a pair of brown eyes, dog-like in their fidelity, and set off well by the frame of smooth, soft brown hair under the neat cap. The air of repose about her is specially marked when compared with the nurse by her side, whose face is more a mask to her feelings than an index to them, and whose alert manner and well controlled nervous excitability show her to be an energetic worker, untiring in her devotion to duty, and also one who can keep her own secrets and other people's too.

There are young faces there, unmarked as yet by this work that does not fail to bring lines and wrinkles to the freshest complexions, while it enlarges the heart and widens the sympathies of the earnest worker: faces with latent possibilities in them—good-looking and plain—thoughtful ones, and here and there a frivolous one but who nevertheless may develop later into one of those who will, after all, perceive something of the inner meaning of life. A touch of delicacy on some of these young faces makes one doubt whether the strenuousness of the work they have chosen may not prove too much for them. Nervousness marks the manner of the newest comer, who only arrived the night, and is in obvious dread of what the day may bring forth. Some—and these are mostly the heads of wards—wear a somewhat anxious and pre-occupied look, and the coming work is evidently uppermost in their minds. The brief meal is soon over, prayers are said, and the nurses disperse to give place in an hour's time to another and smaller group of those who have been on night duty and whose dinner hour it is. These linger over their meal which comes at the end of their night's work, and is partaken of with the leisured ease of those who have nothing before them but recreation and rest.

Ten o'clock, and lo! our dining-room is occupied again. This is luncheon—an informal, picnic sort of affair, to which the nurses come in turns as they have a few minutes to spare from the wards, and at which nobody in particular presides. True, the housekeeper comes in and out, hovering over the table like some amiable, motherly bird of prey, only, instead of swooping down upon the party with intent to devour, she brings sundry dishes from her store-room of all sorts and conditions of things left over from the meals of the day before. Everything gets eaten at luncheon. Odds and ends of pudding and tarts, eggs or cold sausages, sardines—things which were despised at breakfast, or left at dinner, all are devoured with good appetite at this, the pleasantest meal of the day. The dining-room is filled with merry voices and light laughter. Occasionally a lunch is left unfinished, as the door opens and a probationer looks in, with the hurried message, "Doctor's in your ward, Nurse So-and-So," when someone flees, to be on the spot during the morning round; or another arrives, who says to a new probationer, "Sister wants to know why you are so long," whereupon the delinquent, who has probably forgotten all about the time, fares forth with a scarlet face, and hurries back to her ward and the reproof for tardiness which awaits her.

Then the tables are once more laid, this time for the mid-day dinner, and a smaller one is spread with a light repast, in case any of the night nurses should need something more before going to bed. Dinner is divided into two parts, by far the larger number of nurses attending the first half, which is presided over by the matron, and is characterised by the most rigid punctuality. Hospital afternoons are apt to be busy times, and there must be no undue lingering over their meal. The carving is quickly done, and no time is lost in serving everyone. The first dinner is soon merged into the second, and both are supplemented by sundry cups of tea brewed in the ward kitchens afterwards. Then the tables are cleared and re-laid, and the door is shut for an hour or two.

Next, tea-time. This, like dinner, is in two parts; but, unlike that meal, is somewhat irregularly attended. Members of the staff are apt to be in the wards during the afternoons, the out-patient department is crowded, and as likely as not, there are also operations going on in the theatre. Nurses come in for a few minutes as they can spare the time, and

### SKETCHES OF OUR HOSPITAL—*Continued.*

not many can stay for the full half-hour. Sometimes, when the work is not so heavy, tea is a leisurely and pleasant meal, chatted over and enjoyed, often having sundry additions to the regulation diet, in the shape of new-laid eggs, jam, and cake, which of course are welcome. The large, cool, airy room, too, from which the hot sunshine has now gone, is refreshing in its shadiness after some of the sun-baked wards. That is, in summer. In winter, hot pipes running round the walls and a good fire keep it warm and snug.

Half-past eight sees the night nurses at their breakfast, and nine o'clock is the supper hour. The gas is lighted now, and the blinds are down, and some of the faces look tired as they gather round the table. They are all here now, for the night nurses are on duty, and the day staff have finished their work. Supper is a fairly substantial meal, and partaken of by most with an excellent appetite, born of youth, hard work, and a good conscience. There is cheery talk, too, and the prospect of a long night's rest before the duties of the morrow. The faces, on the whole, are happy and contented ones, and pleasant to look upon. They evidently love their work, and do it willingly—"with both hands, earnestly."

Supper over, the signal is given for prayers. Day after day the lofty room echoes to the sweet voices of the nurses in the morning and evening hymns. Led by the little harmonium they sing—as they do most other things at our hospital—as if they liked it; and one or two faces in particular light up with an earnest glow as the words ring out:—

> "Labour is sweet, for Thou hast toiled,
> And care is light, for Thou hast cared."

The familiar old hymn is sung through to the finish, and gains in strength as it goes on, until the last verse ends in a triumph of simple melody, and the sympathetic voices blend tenderly with the little harmonium in its final whisper:—

> "Through life's long day, and death's dark night,
> O gentle Jesu, be our light."

### The Nurses' Bookshelf.

Essentials of Domestic Hygiene or Dangers and Safeguards of Indoor Life. By Clare Goslett. (London: Allman and Son. 1902. Price 2s.)

This is a capital little book, easy reading, and full of useful information. The aim of the author has been to confine herself to essentials, and thus to write a book that should be of service to those who, although wanting those essentials, do not care to overload their minds with the wider and more comprehensive knowledge which is embraced in the so-called science of hygiene, and is to be found detailed in its text-books. Granting a fairly wide interpretation of the word "essential," the author has well succeeded in the mission she has undertaken. To the great majority structural alterations of the dwelling-house are out of the question. As it stands so it must be lived in; for householders who neither know nor care to know anything about domestic hygiene are so plentiful that landlords would generally change tenants rather than incur expense in altering their houses. Hence many pages of this book will probably bring despair rather than encouragement to those who, by force of circumstances, are tied to their present dwellings. That books like this, brightly written, plain, comprehensible and full of common sense, will in the end create, in fact, are already creating, a demand for sanitary houses, is plain enough, but for a long time to come the stolid indifference which exists on the subject of hygiene will make it difficult for the ordinary householder to obtain a dwelling which shall be all that can be desired from a health point of view. A careful study of this book will, however, show the reader how very much can be done to render even very unsatisfactory houses far more safely habitable than they are. The remarks made about the evils of storing rubbish, saving up rags and old papers, and allowing house refuse to accumulate deserve careful consideration, for, convenient as it may be, and indeed is, for those who possess large houses with well-arranged store and lumber rooms, to keep under the roof-tree a general panjorum of odds and ends for use whenever occasion may require, those who have to economise space and labour will often find it better to throw away than to preserve things merely on the theory that they "may come in.' Mrs. Goslett goes a step beyond some writers in her anathema of damp and in the measures she advises for avoiding it. She gives a very good account of the structural defects by which dampness in the house is often caused, but she also points out how largely it may depend on lack of proper domestic management, and how a stuffy house can be rendered habitable by proper attention to aëration, and by care in the washing of floors. She shows how often dampness of rooms, and of nurseries especially, depends upon the bad habit of flooding the floor with water when washing it. There is no doubt she is right in this. Wash the scrubbing brush in plenty of water and as often as may be, but let the water run off it before applying it to the floor. It is not very easy to say anything new on the subject of ventilation, nor perhaps does our author succeed in doing so. She does, however, make a useful point in insisting upon the advantages to be derived from an intelligent use of screens. A folding screen, reaching quite to the ground, should be regarded, we are told, as an indispensable article of furniture in most rooms. It breaks up air currents, and will act like a breakwater in making sheltered spots which can yet be well ventilated; while a screen round a bed at night makes an open window possible under the most difficult of circumstances. On the subject of fresh air we are very properly told that living and sleeping with open windows is largely a matter of habit, and that numerous experiments have proved that open windows can be borne day and night even in the coldest weather, and with the greatest advantage to health, provided individuals are protected by good feeding and woollen clothing. We commend this little book to the careful study of those for whose use it is intended.

The Zenana. Vol. IX. (London: S. W. Partridge and Co.

This volume of "The Zenana" as usual contains a great deal that will be read with interest by all who are engaged in missionary work. Just at present when inoculation against the plague is a great topic of conversation the description given by Miss Aitken of the way the operation is regarded by the natives in the Punjab is worth quoting. "Inoculation," she says "is a gruesome operation. Hypodermic syringe, needle held in spirit lamp, serum injected. Afterwards the arm is swollen, accompanied by pain and fever also frequently great restlessness. All this is much better than having plague, but one can easily see how it impresses the people. They say the needle is made red hot, in this state it is thrust into the arm, and poison injected. And so they fly from inoculation even more than from the plague itself." If this is to be taken as a correct appreciation of native feeling, the prospect of staying the plague by inoculation does not seem very hopeful.

### Everybody's Opinion.

[Correspondence on all subjects is invited, but we cannot in any way be responsible for the opinions expressed by our correspondents. No communication can be entertained if the name and address of the correspondent are not given as a guarantee of good faith, but not necessarily for publication. All correspondents should write on one side of the paper only.]

### A GREAT DISAPPOINTMENT TO POOR LAW PROBATIONERS.

"A Superintendent Nurse of a Poor Law Infirmary" writes: As according to the terms of the new Midwives Act, only one-fourth of the present number of midwifery pupils will succeed in getting their cases and obtaining their certificates and registration under the Central Midwives Board, cannot some compensation be made to that large number of nurses who will find it impossible to enter for their examinations? In Poor Law infirmaries where a large number of puerperal cases are taken in from outside they would obtain their nursing experience in the general wards, and if they attend their 20 cases—quite possibly learning from them as much as the pupil who "personally delivers" them—receive the same instruction theoretically, and pass both a written and an oral examination—made by an independent examiner—of equal severity as that given by the board, obtaining a certificate to that effect from their training school, surely it ought to assist them in obtaining appointments where a knowledge of midwifery is required? I shall be glad to hear the opinion of others on this question. Rule 1, Section C, seems all the harder because some of the medical schools only require their pupils to attend 12 cases, delivering six of them. That the directions given for the routine treatment of the eyes of the newly born are not necessary we have proved at this infirmary, as we have not had a single case of ophthalmia neonatorum in the last 220 confinements.

### THE PENSION FUND AND THE AGE QUESTION.

"Observation" writes: A paragraph in the "Quinquennial Report," just issued by the Secretary of the Royal National Pension Fund for Nurses, in which he speaks of the years "during which a nurse is able to obtain work," must, I think, appeal in a special way to all who are in any way connected with private nursing. From mingling much with nurses engaged in all branches of the work, the conclusion has been forced upon me that it is not so much an age limit that makes it difficult for a large number of private nurses to obtain work as, firstly, a want of elasticity to re-adapt themselves to modern requirements and new conditions under which they work; and, secondly, that attitude of mind in which we find the nurse calculating, as the principal thing, not "How far can I be a help to the people in trouble?" but "Will it be an easy and pleasant case for myself?" I hold that should a nurse arrive at this condition in her career it is time that she should withdraw herself from the ordinary rush of general private nursing and settle down to the care of some old lady or gentleman, or chronic case, where life may be much more monotonous, and salary less, but where she might be very useful and much appreciated, rather than that she should cling to that class of work where she becomes in reality an object of charity. People do not send for a nurse for the pleasure of paying two guineas per week and waiting on her, in return for any little energy she may feel inclined to expend on her patient, and whilst not wishing to make any complaint against the nurse personally they feel she is unequal to the requirements of the case, or as some charitably express it, "A nice woman; I would not like to say anything against her, but either she is not strong enough, or she is getting too old for what we want, and we would rather have someone else next time." Does it not, therefore, behove us all to aim for such provision that, should a time come when we feel out of harmony with our work, we may have something to fall back on, and so supplement the smaller salary which we may be able to obtain by accepting only such work as we can honestly perform to the comfort of others and with credit to ourselves, and not be tempted to keep to work we are not quite equal to for the sake of the larger salary?

### Appointments.

Ashbourne Cottage Hospital.—Miss Annie Smith has been appointed nurse-matron. She was trained at the Royal Southern Hospital, Liverpool, and has since been assistant nurse at Wantage Cottage Hospital, assistant nurse at Loughborough General Hospital, nurse at Boston Hospital. She has also taken matron's holiday duty at Mount Sorrel Cottage Hospital.

Brighton Workhouse Infirmary.—Miss Jessie M. Akehurst has been appointed charge nurse. She was trained at Brighton Workhouse Infirmary, and has for the last six months been doing holiday charge work. She holds the L.O.S. certificate.

Burnley Union.—Miss Sarah Taylor has been appointed charge nurse. She was trained at Chorlton Union Infirmary, where she was afterwards charge nurse. She holds the L.O.S. certificate.

Lewes Home for Inebriates.—Miss Sara Burnside Boyd has been appointed assistant matron. She was trained at Birmingham Infirmary, and has since been charge nurse at Brighton Workhouse Infirmary. She holds the L.O.S. certificate.

Newcastle-on-Tyne Union Hospital.—Miss Margaret Keohane has been appointed charge nurse. She was trained at the Hackney Infirmary, Homerton, where she has since done staff and charge duties. She holds the L.O.S. certificate.

Paddington and Marylebone District Nursing Association.—Miss S. M. Marsters has been appointed superintendent. She was trained at the Norfolk and Norwich Hospital, where she was afterwards charge nurse. She has since been matron of the Mildenhall Cottage Hospital, district nurse for the North London Nursing Association, and superintendent of the Hampstead District Nursing Association.

Stirling District Asylum, Larbert.—Miss Annabel L. Irvine has been appointed matron of the male department. She was trained at the Western Infirmary, Glasgow, where she was afterwards sister, and at the Perth District Asylum, Murthly. For the last five years she has been matron of the Royal and District Asylum, Dundee.

### Presentations.

Bristol Royal Infirmary.—On leaving the Bristol Royal Infirmary to take up her duties as matron of the Cumberland Infirmary, Carlisle, Miss Cummins was presented with a silver James I. teapot by the nursing staff, a silver sugar basin matching from the matron, while the maids in the house gave a handsome cushion and the laundrymaids a picture, the subject being a copy of "Dante's Dream," by Rossetti.

### Wants and Workers.

The Hospital.—Will any nurse forward The Hospital the Monday after publication in return for prepaid postage? (stamped addressed wrapper). Nurse Sims, Cottage Hospital, Brackley.

Disused Garments.—Will any kind friend having any disused children's or other garments, old shoes, or stockings to spare, no matter how old or worn, send them to District Nurse, Haveringland, near Reepham, Norfolk. Haveringland is a very large, poor district with many families of six and eight small children. Men's wages are 12s. per week. Reference to rector of parish if required.

### Echoes from the Outside World.

### The King's Memorial to Queen Victoria.

ON Sunday the King unveiled a memorial to Queen Victoria in the parish church at Crathie. In addition to his Majesty, who was in Highland dress, there were present at the service and subsequent interesting ceremony the Prince and Princess of Wales, Princess Charles of Denmark, and Prince Edward of Wales. At the conclusion of the service by the Moderator of the Church of Scotland, Dr. Gillespie, who paid an eloquent tribute to the personal piety of Queen Victoria and her large-heartedness, a hymn was sung, and after a short prayer the King rose from his seat, and, advancing a step, pulled the cord connected with the draperies veiling the bust, and on its being uncovered returned at once to his seat. Then followed the dedication by Dr. Gillespie, and the Benediction. The monument consists of a portrait bust in white statuary marble, and is placed in a granite niche on the face of one of the granite pillars which support the arches of the chancel and the Royal transept. It was at the base of this pillar that the foundation stone of Crathie church was laid by Queen Victoria with her own hands in 1893. The Queen is represented as wearing the crown with a veil. She also wears State robes and the Order of the Thistle. On the monument is the following inscription:—"In dutiful and beloved remembrance of Victoria, Queen of Great Britain and Ireland, Empress of India, this monument was erected by her sorrowing and devoted son, Edward R. and I." The base of the monument bears the letters "V. R." The bust was the work of the well-known sculptor, Herr Emil Fuchs, and the niche, canopy, and bracket of the architect Mr. A. Marshall Mackenzie of Aberdeen. In the niche and canopy full advantage has been taken of the variety of colours in the granite of Aberdeenshire. Two mural tablets, respectively to the memory of the Duke of Saxe-Coburg and Gotha and the Empress Frederick, have also been erected by the King, one each side of the Royal pew, with inscriptions by his Majesty.

### Movements of Royalty.

THE German Emperor brought his visit to Vienna to a close on Sunday evening, taking leave of the Emperor Francis Joseph, with whom he had driven from Schönbrunn, at the station. On Saturday the Austrian Heir-Apparent and the German Emperor went deer-shooting, and in the evening both the Emperors were entertained at the German Embassy in Vienna, his Majesty the Emperor Francis Joseph having unexpectedly invited himself. As the Emperor William entered the Embassy he received from Count Wedel a gold medallion portrait of the Emperor Francis Joseph mounted on a small block of marble. It is the work of the court sculptor, and was presented as a souvenir. On Sunday morning the Emperor William attended service in the Evangelical Church; in the afternoon he was present at a banquet to 150 guests in the large gallery at Schönbrunn; and early in the evening, before his departure, he witnessed a dramatic performance in the theatre. On Monday, the German Emperor proceeded to Dantzig, and unveiled a monument to the memory of Emperor William I., proceeding afterwards to the military headquarters, where he delivered a stirring address to the Germans employed in the different workshops.

### The Cabinet Crisis.

THE meetings of the Cabinet last week were followed by the announcement that several Ministers had resigned office in consequence of differences of opinion on fiscal policy. Mr. Ritchie has ceased to be Chancellor of the Exchequer, Lord George Hamilton is no longer Secretary of State for India, and Lord Balfour of Burleigh has relinquished the post of Secretary for Scotland. But far more important than either of these, or than all of them put together, is the fact that Mr. Chamberlain has withdrawn from the office of Secretary of State for the Colonies. His retirement from the Government has overshadowed every other topic since it was signified, and there are strong doubts as to whether the Administration can survive his departure after Parliament meets. The essential distinction, however, between his resignation and that of the other ex-Ministers is that while they are understood to be totally opposed to any interference with the existing fiscal system and therefore entirely out of sympathy with the Prime Minister's declaration in favour of the imposition of retaliatory duties, Mr. Chamberlain will continue to loyally support the Government which he has only left in order that he may be free to pursue his campaign as an advocate of preferential tariffs.

### Terrible Accident at Scafell.

ON Monday afternoon four tourists were attempting to climb what is known as the pinnacle of Scafell, when all of them fell and sustained injuries which proved immediately fatal in the case of three of the party, while the fourth died in the course of being carried down to Wastwater Hotel. The names of the victims are Henry L. Jupp, Wellesley Road, Croydon; Stanley Ridsdale, Hatherley House, Kew Gardens; A. E. W. Garrett, Ross Road, Wallington; and R. W. Broadrick, Windermere, a master at Fettes College, Edinburgh. The ascent which the ill-fated party endeavoured to make is recognised by climbers as the most difficult piece of ground on Scafell. The first attempt to climb it was made in 1887 by a member of the Alpine Club, who failed, but eleven years later succeeded, and was killed a year or two subsequently when climbing in Switzerland. The late Professor Marshall, of Owens College, Manchester, lost his life on Scafell a few years ago within 100 yards of the spot where the accident which is now recorded occurred. The height of the climb to the pinnacle is about 600 feet.

### Trip of an Airship over London.

AFTER two or three disappointments owing to unfavourable wind or weather, Mr. Stanley Spencer made a successful trip with his airship on Thursday last week. About half-past four the huge machine was brought out from the shed where it was housed at the lower end of the Crystal Palace gardens, and after twice having to return to earth—first because of faulty balance and then because of a slight difficulty with the motor—the ship rose straight into the air, coming into contact as it moved with a telegraph wire which it snapped at once. Mr. Spencer steered round the north tower, and after making one or two complete circles the ship headed for the City. For some time after it had mounted the yellow gas-holder of the ship was visible against the clouded sky, and amongst those who watched till she was out of sight were the wife and little child of the aeronaut. An enormous number of people had assembled as early as three o'clock in the neighbourhood of St. Paul's, as Mr. Spencer had announced his intention of going round the Cathedral. It was half-past five before she was seen by the crowd and it soon became evident that the wind was too strong to allow of Mr. Spencer either circling the dome as he had hoped, or returning to the Crystal Palace, and he speedily sailed away in the direction of the Alexandra Park, the alternative spot chosen for disembarking. He ultimately descended safely in Trent Park, New Barnet. The inventor subsequently stated that the current was so strong round St. Paul's that the propellor only revolved 80 times a minute instead of 300, and therefore he could make no headway against the wind.

### For Reading to the Sick.

" *He shall give His angels charge over thee*

*To keep thee in all thy ways.*"—Psalm xci. 11.

They are evermore around us though unseen to mortal sight,
In the golden hour of sunshine, and in sorrow's starless night,
Deepening earth's most sacred pleasures with the sense of sin forgiven,
Whispering to the lonely mourner of the painless joys of heaven.

Lovingly they come to help us, when our faith is cold and weak,
Guiding us along the pathway to the Blessed Home we seek:
In our hearts we hear their voices, breathing sympathy and love,
Echoes of the spirit-language in the sinless world above.

They are with us in the conflict with their words of hope and cheer,
When the foe of our salvation and his armèd hosts draw near;
And a greater One is with us, and we shrink not from the strife,
While the Lord of angels leads us on the battlefield of life.

*Anon.*

Think, O my soul, in what marvellous ways the Holy Angels not only guard but help, comfort, soothe, lift me. It is sweet to be in good company. Sweet to be among those who think noble thoughts, and say gentle, strong, brave, pure words. Why, amidst those who are evil, and fear not God, do there come to me sweet thoughts, kind and humble towards sinners, but strong and helping against sin? Why, amidst base and evil speakers, comes the whisper, "Blessed are the pure in heart, for they shall see God"? My Angel, who knows something of the exquisite beauty of that vision, is reminding me, lest I should lose that joy. Why do there come sudden, sweet thoughts of holiness and goodness, and a noble life, and better things, when I hear sweet music, noble poetry, watch a stately sunset or a tender dawn? My Angel is helping me to enter into the meaning of the lovely things which are a joy to him. When I come to die, may I be comforted by the thought of the loving and Blessed Ones, waiting to bear my soul to God.

Remember how the Holy Angels watched over, guarded, cared for our Lord, in His human life. These Blessed Ones comforted my Lord in His temptation; if I allow them, they will comfort me. One strengthened my Lord in His penance of blood in Gethsemane; if I allow them, they rejoice in my repentance.—*Canon Knox Little.*

All our days direct us
In the way we go,
Lead us on victorious
Over every foe:
Bid Thine Angels shield us
When the storm-clouds lour,
Pardon, Lord, and save us
In the last dread hour.

*Rev. T. J. Potter.*

### Notes and Queries.

**FOR REGULATIONS SEE PAGE 307.**

*Home.*

(267) Will you tell me of any home, public or private, where a young woman of 39, slightly feeble in intellect, but healthy, could be received, with a view to her residence being permanent?—*I. F. M.*

Consult the Secretary, the National Association for Promoting the Welfare of the Feeble-Minded, 53 Victoria Street, S.W.

*Invitation.*

(268) Will you kindly tell me if having been trained at the District Nurses' Home, Plaistow, holding the L.O.S., and also having been employed as district nurse for four years, entitles me to an invitation for any nurses' function?—*N. J. F.*

You would be eligible for an invitation to any social gathering designed to include district and maternity nurses.

*Dispensing.*

(269) Will you tell me if there is any school or chemist in Oxford where I could train for the Apothecaries' Hall certificate?—*A. M.*

Apply to the Secretary, the Pharmaceutical Society, 17 Bloomsbury Square, W.C.

*Johannesburg Hospital.*

(270) Can you tell me if the nursing staff of the Johannesburg Hospital is Roman Catholic? What is meant by lay-sister?—*A. A.*

Yes. A lay-sister is one who is not a professed réligieuse.

*Paris.*

(271) Will you inform me if there are any private asylums in Paris where good mental paying patients (English) are received? I should like to obtain employment there if I can.—*A. H.*

Possibly the matron of the Hertford British Hospital, Rue de Villiers-Levallois-Perret, Paris, would kindly advise you. We do not give the names of private institutions.

*M.B.*

(272) Are there any means of study I could adopt whilst following my duties as a private nurse, for the degree of M.B.? Unable through ill-health to obtain my certificate, I should like to employ my spare time studying for a degree.—*Student.*

Apply to the Secretary, the London School of Medicine for Women, 8 Hunter Street, Brunswick Square, W.C.

*Mental Case.*

(273) Will you tell me how to obtain a mental case? I am anxious to take one into my home.—*C. E. P.*

Inform all the medical men of your acquaintance of your wish, and advertise in the medical journals.

*Melbourne or Sydney.*

(274) Will you be kind enough to give me the addresses of one or two nursing homes in Melbourne or Sydney? Can you give any particulars of monthly, private, and district nursing, and also tell me how to obtain introductions to medical men?—*M. S. M.*

The Melbourne Nurses' Home, 6 Parliament Street. Resident nurses pay £1 1s. per month, and 15s. a week for board when in residence. The outdoor staff pay 5 per cent. on all work obtained through the home. Fees from £2 2s. per week. You can only obtain introductions through mutual acquaintances.

*Abroad.*

(275) I am anxious to take up private nursing on the continent in the autumn. I find, on application, that the Nice Institute requires a three years' training, and I have had only two years. I should be grateful if you could tell me of any institutes to which I could apply.—*Irene.*

A three years' certificate is required by most, if not by all, nursing institutes abroad. A list will be found in "Burdett's Hospitals and Charities."

**Important Nursing Textbooks.**

"The Nursing Profession: How and where to Train." 2s. net; 2s. 4d. post free.

"A Handbook for Nurses." (New Edition). 5s. net; 5s. 4d. post free.

"The Human Body." 5s. post free.

"Ophthalmic Nursing." (New Edition). 3s. 6d. net; 3s. 10d. post free.

"Gynæcological Nursing." 1s. post free.

**"Art of Feeding the Invalid." (Popular Edition). 1s. 6d. post free.**

**"Practical Hints on District Nursing." 1s. post free.**

